# Understanding intra-individual isotopic variability in modern cremated human remains for forensic and archaeological studies

**DOI:** 10.1371/journal.pone.0320396

**Published:** 2025-04-24

**Authors:** Christophe Snoeck, Melanie M. Beasley, Dawnie Wolfe Steadman

**Affiliations:** 1 Archaeology, Environmental Changes & Geo-Chemistry, Vrije Universiteit Brussel, Brussels, Belgium; 2 G.-Time Laboratory, Université Libre de Bruxelles, Brussels, Belgium; 3 College of Liberal Arts, Purdue University, West Lafayette, Indiana, United States of America; 4 Forensic Anthropology Center, University of Tennessee, Knoxville, Tennessee, United States of America; University of Padova: Universita degli Studi di Padova, ITALY

## Abstract

Cremated bone fragments can be studied using structural, elemental, and isotope analyses in archaeological contexts to reconstruct funerary practices and understand past mobility and migrations of populations that practiced cremation. However, the potential of isotope analyses of cremated bone in forensic contexts remains heavily unexplored. The identification of fire victims can be complex as the remains can be extremely fragmented and commingled. The high temperatures (up to 1000°C and above) destroy most organic matter such that, obtaining reliable DNA from such intensively burned human remains is extremely difficult. Still, other signals present in bone, such as strontium concentrations and isotopes, are preserved during cremation, and could be used to assess the geographical origin of unidentified fire-affected individuals. Carbon and oxygen isotope ratios together with infrared analyses provide information about the burning conditions and could help understanding how a body was burned. Here, isotope and infrared analyses are carried out on fourteen recently deceased cremated individuals of known residential history from the UTK Donated Skeletal Collection curated by the Forensic Anthropology Center (Knoxville, Tennessee). By carrying out these measurements on different bones with different turnover rates (i.e., otic capsule of the petrous part of the temporal bone, femur, and rib), we endeavor to reconstruct life histories of recently deceased cremated individuals and gain new insights into cremation practices. The results highlight differences in carbon and oxygen isotopes between different skeletal elements and confirm their potential to gather information about the way a body was burned (e.g., temperatures, fuel used). Strontium concentrations and isotope ratios were also measured to assess the geographical origin of these individuals. The use of strontium isotope ratios, however, seem to have limitations for individuals born in the last few decades due to globalization of consumed food resources. Nevertheless, it is still possible to obtain information about the birthplace of older individuals (> 50 years) by analyzing strontium isotope ratios in the petrous part of their temporal bone, which retains a signal linked to the first few years of their lives when local resources were still used in larger quantities compared to today.

## Introduction

Lanting & Brindley [1] demonstrated that reliable radiocarbon dates could be obtained from calcined bone, ushering in a new era for the study of cremated bone in archaeological contexts. In 2015, another step occurred that boosted the importance of calcined bone in archaeological and forensic studies. It was demonstrated that any type of calcined bone (not only the otic capsule of the petrous parts of the temporal bone [2]) could provide a reliable substrate for strontium isotope analyses [3] to identify the possible geographical origin(s) of cremated individuals for palaeomobility studies and the identification of modern burned human remains in forensic contexts. Indeed, due to its much higher crystallinity compared to unburned bone, fully calcined bone (white bone burned at temperatures above 650°C) is more resistant to post-burial strontium exchanges [3]. Furthermore, the strontium concentrations in bioapatite are unaffected by burning (see [4] for more details). This development allows for palaeomobility studies in times and places where cremation was practiced (e.g., [5–20]) and potentially in forensic cases when DNA extraction is not possible [21,22], though its use has remained limited.

Strontium isotope ratios (^87^Sr/^86^Sr) measured in human and animal bone and teeth record mainly the origin of their foods, which take up their strontium from the lithological composition of the soils in which they grew. By measuring ^87^Sr/^86^Sr, it is possible to assess if individuals lived (i.e., consumed food) close to their burial place or not, and which parts of the landscape they were using. However, in archaeological contexts, these measurements are only possible on unburned teeth and calcined bone. Unburned bone, due to its lower crystallinity and higher organic content compared to tooth enamel and calcined bone, will exchange strontium with its burial environment which strongly affects the endogenous signal [23,24]. Even tooth enamel can, in some cases, be affected by diagenesis (see [25–27]).

In a Neolithic circular chamber at Ballynahatty, Co. Down (Northern Ireland), for example, both unburned skulls and cremated bone were discovered. ^87^Sr/^86^Sr showed that cremated individuals were consuming foods from the south and/or west of the site while unburned individuals used parts of the landscape to the north of Ballynahatty. This demonstrated that cremated and unburned individuals who had used different parts of the landscape in life were buried within the same circular chamber [5]. At Stonehenge, the single largest Late Neolithic burial site known in Britain [28], the results obtained directly from 25 calcined cranial bone fragments showed that at least 40% (10 out of 25) of those buried at Stonehenge came from much further afield, perhaps as far as West Wales (> 200 km), which is the source of the blue stones used to build part of the monument [6].

Combining strontium isotope ratios and strontium concentration measurements ([Sr]) offers the opportunity to look into dietary practices [29]. [Sr] provides information on relative importance of animal and plant foods in the diet, as well as marine resources [30,31], as modern-day seawater and, therefore, marine resources have a specific ^87^Sr/^86^Sr of 0.7092 [32]. Marine resources also have a generally higher strontium concentrations compared to terrestrial resources [4]. For example, two Mesolithic cremated bone fragments from Langford (Essex, England) had strontium concentrations higher than 300 ppm and ^87^Sr/^86^Sr close to 0.7092, suggesting the consumption of significant amounts of marine resources [33]. These results show how [Sr] can be used to look at the diet of past populations that practiced cremation (as all organic matter, including collagen, usually used for such studies is destroyed) as well as the use of salt [4], though more work is required to better understand how [Sr] vary within an individual and across the landscape.

It is also important to keep in mind that bone continues to remodel throughout life, and so provides information relating to the later years of adult life depending on the skeletal element analysed [34,35]. This needs to be contrasted with traditional measurements carried out on unburned tooth enamel which relate to tooth crown formation time and reflect childhood to adolescence dietary intake. Overall, rib remodels rather fast (a few years to a decade), reflecting the final years of an individual’s life, while femur has a much slower turnover rate [34]. Nevertheless, most of this work has focussed on carbon isotopes in collagen and more work is needed to evaluate the turnover rates of other relevant chemical elements such as strontium, to enable the reconstruction of life histories. A recent study focussing on strontium isotopes highlighted that because the petrous part of the temporal bone does not remodel, a unique signal linked to the first few years of an individual’s life is preserved [36].

While [Sr] and ^87^Sr/^86^Sr measured in calcined bone provide insights on the geographical origin and diet of burned individuals, the combination of infrared analyses and carbon (δ^13^C) and oxygen (δ^18^O) isotope ratios measured on the carbonate fraction of bone apatite provide insight as to how a body was burned (i.e., temperature, type of fuel, oxygen availability, etc.). Contrary to unburned skeletal remains, where δ^13^C and δ^18^O values of the carbonate fraction of bone apatite are used to study diet and origin of drinking water, respectively, the carbon and oxygen isotope composition of burned bone reflect a mixture of original carbon and oxygen mixed with carbon and oxygen originating from the combustion atmosphere [37–40]. Infrared analyses provide information specifically about the temperatures at which bodies were burned [40–44]. It also allows the detection of cyanamide that has been suggested to be present in bones burned under more reducing conditions, e.g., when oxygen availability is limited [38,40,44,45].

The use of these isotope ratios and infrared indices has significantly increased the amount of information that can be obtained from burned human remains in both archaeological and forensic contexts [46]. However, many questions remain about the variability in isotope ratios and infrared indices within a single individual, their link to the life histories of individuals, and the way cremation took place (temperature, fuel used, position of the body, presence of clothes, etc.). The University of Tennessee, Knoxville, Donated Skeletal Collection curated at the Forensic Anthropology Center (Knoxville, Tennessee) offers a unique opportunity to deepen our understanding of the elemental, structural and isotopic variability within a single cremated individual. Here we study three different skeletal elements (petrous part of the temporal bone, femur, and rib) of fourteen individuals cremated between 2010 and 2016 using δ^13^C, δ^18^O, ^87^Sr/^86^Sr, [Sr] and infrared analyses.

## Materials and methods

### Samples, sampling and pre-treatment

Fourteen individuals aged between 47 and 85 years, from the UTK Donated Skeletal Collection cremated between 2010 and 2016 were sampled for the analyses (Table 1). This study was possible because of the donation of cremains to the UTK Donated Skeletal Collection at the Forensic Anthropology Center. Each individual donor or family consented to the donation of their remains to the collection and for the use of their remains for education and research.

**Table 1:** List of individuals with place of birth and death.

Individual	Place of birth	Place of Death	Other residence*
1	**England**	Columbus, OH	
2	Moline, IL	Bristol, VA	Abingdon, VA
3	Bristol, MI	Lynnville, MI	
4	Dalton, GA	Dalton, GA	Las Vegas, NV
5	Detroit, MI	Lincoln Park/Westland, MI	Las Vegas, NV
6	Chicago, IL	Salt Lake City, UT	Los Angeles, CA
7	Boaz, AL	Boaz, AL	Madison, AL
8	**Argentina**	Cornelius, NC	Miami, FL
9	Wilmington, DE	Chesterfield, VA	Midlothian, VA
10	West Palm Beach, FL	Mammoth Cave, KY	Okeechobee, FL
11	Cumberland, MD	Richmond, VA	
12	Hinsdale, IL	Cleveland, TN	
13	Danville, VA	Charleston, TN	
14	Oakland, MI	Decatur, TN	

*If moved less than 10 years before death.

For each individual, a fragment of rib and a fragment of femur were selected. Additionally, the petrous part of the temporal bone was sampled using a diamond drill. This was, however, done before the study of Veselka et al. (2021b) who demonstrated the need to first cut the petrous part along the midmodiolar plane. This means there is a possibility that we sampled some bone that does not belong to the inner cortex, which is the only part that does not remodel. Two ribs (8 & 10), two femora (2 & 6) and eight petrous parts (1, 6, 7, 8, 11, 12, 13 & 14) were sampled twice to assess intra-bone variability. In the case of the petrous part, the first sample was comprised of almost exclusively the inner cortex of the otic capsule of the petrous part of the temporal bone with a very limited amount of surrounding bone, while the second sample was mostly composed of surrounding bone.

The fourteen studied individuals were burned in ten different facilities. Three individuals (2, 9 and11) were cremated in the same facility (*Crematorium A*) while three others (12, 13 and 14) were burned in *Crematorium B*. The other seven individuals were cremated in different crematoria. Most modern cremation retorts are fueled with natural gas [47]. All individuals were born, lived and died in the US, except Individuals 1 and 8 who were born in the UK and Argentina respectively, but lived their later years and died in the US (Table 1). The donors were of both sexes and aged between 47 and 85 years old when they died.

Prior to analyses, each of the 54 calcined bone fragments were rinsed three times with milliQ water. For each rinsing, the samples were placed for 10 minutes in an ultrasonication bath. Calcined bone fragments were treated with 1M acetic acid for 3 to 10 minutes in an ultrasonication bath and then rinsed again three times with milliQ water and 10 minutes ultrasonication [3,48]. The samples were then left overnight in an oven at 50°C. Once dry, they were crushed using a mortar and pestle.

### Fourrier transform infrared spectroscopy (FTIR) analyses

Calcined bone powder was analysed using FTIR in Attenuated Total Reflectance (ATR) mode on a Vertex 70v infrared spectroscope (Bruker, Kontich, Belgium). Each sample was pressed onto a diamond crystal and placed under vacuum before measurement. The background was measured and removed, and a baseline correction was carried out using the Opus software. Each sample was measured in triplicate and the results of the various calculated ratios averaged. The infrared ratios used here are API, BPI, C/C, IRSF, OH/P and CN/P (see [44] for more details). Due to its ability to work under vacuum, the Vertex 70v can clearly detect low levels of cyanamide with CN/P ratio of 0.02 (see [45]) and above contrarily to the instrument used in [44] that could only clearly detect cyanamide when CN/P > 0.25.

### Carbon and oxygen isotope analyses

Without further pre-treatment, approximately 2mg of calcined bone powder were placed in glass vial and the carbon and oxygen isotope ratios (reported in VPDB) measured on a Nu Perspective Isotope Ratio Mass Spectrometer (IRMS) with a NuCarb carbonate preparation device at the Vrije Universiteit Brussel (VUB, Brussels, Belgium). Internal standards MAR2, ENF and CBA were used (see [49] for more details) as well as international standards IAEA-CO8, IAEA-603, NBS18 and Iso-Analytical IA-R022. For the internal calcined bone standard CBA, the reproducibility (n = 37; 1SD) is 0.40 and 0.42‰ for δ^13^C_ap_ and δ^18^O_C_ respectively.

### Strontium isotope analyses

About 15mg of calcined bone powder was then used for strontium isotope analyses, placed in a Teflon beaker and digested in 1mL of subboiled 14M HNO_3_. After complete dissolution, the samples were left to dry on a hotplate at 100°C until dryness. Strontium was extracted from the samples and purified following the protocol described in [3] and measured on a Nu Plasma MC-ICP Mass Spectrometer (Nu015 from Nu Instruments, Wrexham, UK) at the Université Libre de Bruxelles (ULB). During the course of this study, repeated measurements of the NBS987 standard yielded ^87^Sr/^86^Sr = 0.710246 ± 0.000045 (2SD for >300 analyses), which is consistent with the mean value of 0.710252±13 (2SD for 88 analyses) obtained by TIMS (Thermal Ionization Mass Spectrometry) instrumentation (Weis et al. 2006). All the sample measurements were normalised using a standard bracketing method with the recommended value of ^87^Sr/^86^Sr = 0.710248 [50]. Procedural blanks were considered negligible (total Sr (V) of max 0.02 versus 7–8V for analyses; i.e., ≈ 0.3%). For each sample the ^87^Sr/^86^Sr value is reported with a 2SE error (absolute error value of the individual sample analysis – internal error).

### Strontium concentration analyses

Sr and Ca concentrations in the sample digests were determined using a Thermo Scientific Element 2 sector field ICP mass spectrometer at the Vrije Universiteit Brussel (VUB), Belgium, in low (^88^Sr) and medium (^42^Ca) resolution using Indium (In) as an internal standard. An external calibration was carried out using different matrix matched reference materials (SRM1400, CCB01). The actual strontium concentrations were then normalized to 40wt.% Ca. Accuracy was evaluated by the simultaneous analysis of two internal bioapatite standards (ENF and CBA). Based on repeated digestion and measurement of these reference materials, the analytical precision of the procedure outlined above is estimated to be better than 5% (1SD, n = 33 for CBA and n = 5 for ENF).

### Statistics

All datasets were tested for normality using a Shapiro-Wilk Normality Test [51], and a non-parametric test, a Wilcoxon rank sum test [52], was used to compare data classes in R Studio 2023.06.1. A p-value, and test statistic where necessary, is reported for each statistic, and the significance level is set at 0.05 for all analyses.

## Results

The detailed results of the various analyses can be found on Zenodo [53]. For the rib and femur duplicate samples, the difference in strontium concentrations ([Sr]) and isotope ratios (^87^Sr/^86^Sr) are extremely small (Δ[Sr] ≤ 5 ppm; Δ^87^Sr/^86^Sr ≤ 0.0002). Carbon (δ^13^C) and oxygen (δ^18^O) isotope ratios are much more variable with a difference of up to 2.5‰ and 5.4‰, respectively. This can be expected as the strontium present in calcined bone represents an average of the dietary intake of strontium over several years/decades [3]while the carbon and oxygen isotope ratios reflect the very variable cremation conditions [40,54]. In the case of the petrous parts, the differences in [Sr] and ^87^Sr/^86^Sr are much larger, rising to 25 ppm and 0.0015, respectively. δ^13^C and δ^18^O values also vary immensely with variations of up to 5.1‰ and 4.6‰, respectively. In view of the strontium results, the two different samples taken from and around the otic capsule of the petrous part do not represent the same strontium intake. Indeed, only the otic capsule of the petrous part of the temporal bone preserves a unique signal linked to the first few years of an individual’s life while the surrounding bone does not. Extreme care in sampling is therefore crucial and requires to follow the procedure outlined in [36]. From this, the second samples taken around the petrous part are excluded from this study. The results obtained for two fragments of the same rib or femur are averaged.

The distribution of each δ^13^C dataset; petrous part, rib and femora, was examined. The petrous part dataset normally distributed (Shapiro-Wilk W = 0.85287, p-value = 0.0243), while the femora and rib were not normally distributed (W = 0.98381, p-value = 0.9912 and W = 0.94608, p-value = 0.5017 respectively). As two datasets are not normally distributed a non-parametric test, the Wilcoxon rank sum test was used to compare these datasets.

**Fig 1:**
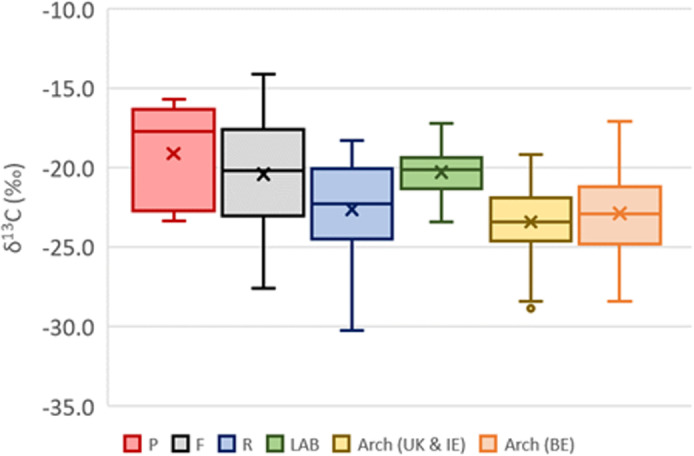
Carbon isotope ratios of the different skeletal elements (P = petrous part; F = femur; R = rib) compared to cow tibia fragments heated in a muffle furnace (LAB [40]) and archaeological cremated bone fragments from Ireland [5,7], the UK [6], and Belgium [54].

**Fig 2:**
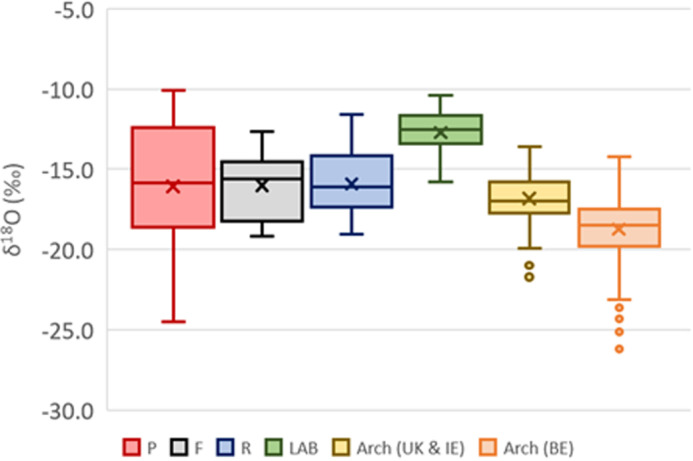
Oxygen isotope ratios of the different skeletal elements (P = petrous part; F = femur; R = rib) compared to cow tibia fragments heated in a muffle furnace (LAB [40]) and archaeological cremated bone fragments from Ireland [5,7], the UK [6], and Belgium [54].

The carbon isotope ratios (Fig 1) vary between the different skeletal elements and while the difference between the petrous parts (P) and the femora (F) and between the femora (F) (p= 0.4013, W = 117) and the ribs (R) (p-value = 0.1936, W = 127) are not statistically significant, the difference between P and R is (p-value = 0.0106, W = 153). When comparing this data with previously published studies, the δ^13^C values appear closer to those seen in a modern cow tibia heated in a muffle furnace (LAB; n = 46 [40]) than those measured in 71 archaeological human remains from Ireland [5,7] and the UK [6] or 207 from Belgium [54]. The oxygen isotope ratios (Fig 2) show a different trend with similar median values for all three groups of skeletal elements. All δ^18^O datasets; rib, femora, and petrous parts are not normally distributed (all p-value > 0.3762). Comparing each dataset indicates that there are no statistically significant differences between skeletal elements (all p-values > 0.7345). An opposite pattern is observed with the δ^18^O values of the different skeletal elements being closer to those measured in archaeological cremated human remains than those of the cow tibia heated in a muffle furnace. The interquartile ranges observed for both carbon and oxygen isotope ratios are much higher in the modern cremated human remains than in the experimentally burned animal samples and the archaeological cremated human remains. This range is even larger in the petrous parts than in the femora and ribs (Fig 1).

**Fig 3:**
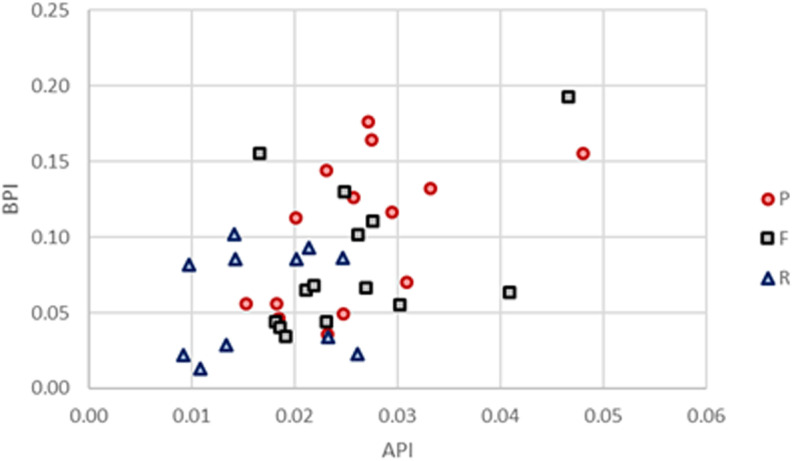
API vs BPI for the petrous parts, femora and ribs.

**Fig 4:**
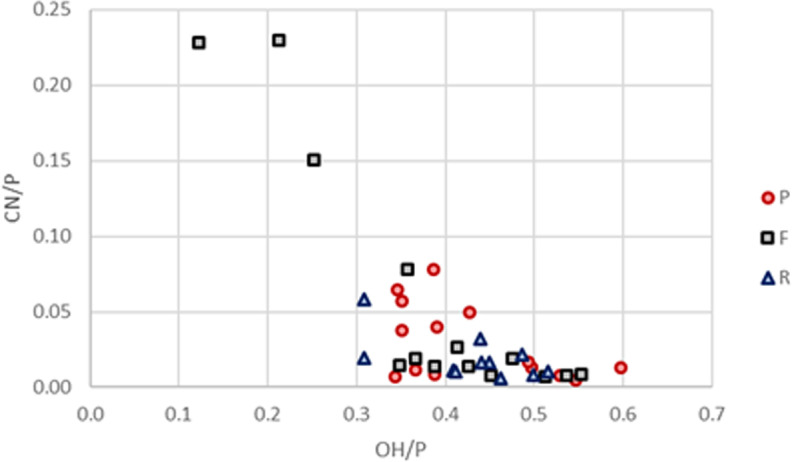
OH/P vs CN/P for the petrous parts, femora and ribs. Note that the three highest CN/P values belong to three different femora (8, 13 & 14).

The infrared results confirm that all samples are fully calcined with low amounts of carbonates left (BPI and API) and high crystallinity (IRFS values between 3.7 and 6.2). The range of IRSF values is slightly lower than observed in calcined cow tibia fragments heated in a muffle furnace at temperatures above 600°C (4.2 to 6.7 [44]). Ribs have generally lower carbonate content than femora and petrous parts with the difference between ribs and petrous parts being statistically significant (Wilcoxon signed rank, W=4, p-value=0.0068), and the difference between ribs and femora being almost significant (W=12, p-value=0.06738; Fig 3). According to the C/C and IRSF values, the samples were burned between 800 and 900°C but the comparative baseline used [44] is limited to temperatures up to 900°C. It is very likely that the temperatures could be higher, up to 1000–1100°C, in crematoria. The CN/P values are generally low (< 0.10) except for three femur samples with values between 0.15 and 0.25 (Fig 4) suggesting the individuals were burned under oxidizing conditions.

Interestingly, when comparing the carbon isotope ratios measured on bones burned in different crematoriums, those burned in *Crematorium A* are generally enriched in ^13^C compared to those from *Crematorium B*, with a difference of 4.5‰ between the medians of the two groups of 9 bone fragments (though Wilcoxon rank sum test, W = 103, p-value = 0.07802). No such trend is observed in the oxygen isotope ratios. Furthermore, no difference can be seen in the various infrared indices except for the CN/P values that are much more variable and generally higher in the bones burned in *Crematorium B*. They have values ranging from 0.005 to 0.228 with a median value of 0.017 compared to *Crematorium A* where the CN/P values range from 0.007 to 0.057 with a median value of 0.008 (though Wilcoxon rank sum test, W = 35, p-value = 0.193).

**Fig 5:**
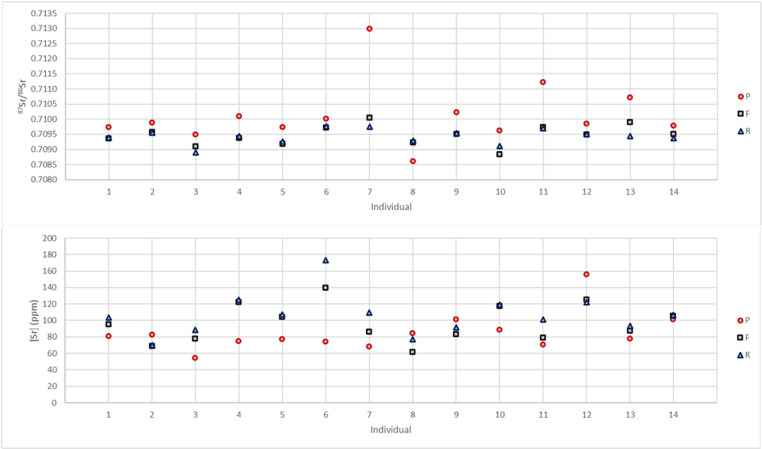
(Top) 87Sr/86Sr and (bottom) [Sr] for the petrous parts, femora, and ribs of each individual (P = petrous part; F = femur; R = rib). Sr concentrations are normalized to 40wt.% Ca.

**Fig 6:**
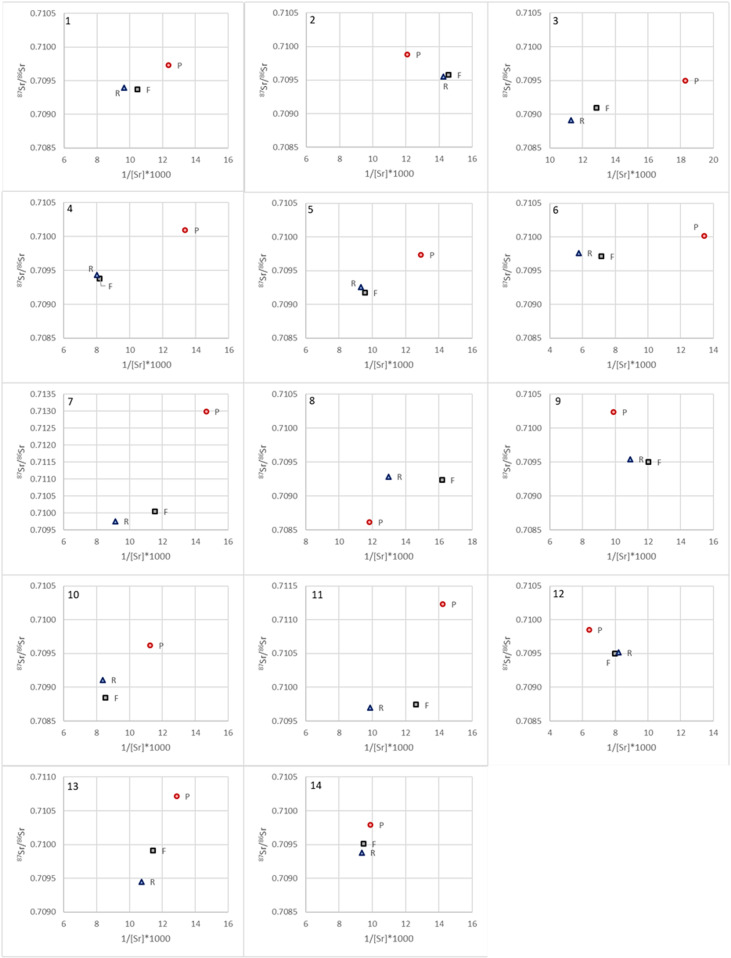
^87^Sr/^86^Sr vs 1/[Sr]*1000 for each bone fragment of each individual (P = petrous part; F = femur; R = rib); note all axes have the same range except for individual 7. Sr concentrations are normalized to 40wt.% Ca.

The strontium isotope ratios of the petrous parts, femora and ribs range from 0.7086 to 0.7130 while [Sr] vary from 55 to 173 ppm. [Sr] and ^87^Sr/^86^Sr show differences between different skeletal elements of a single individual going up to 0.0032 (Individual 10) and 99 ppm (Individual 6). In most cases, the difference in ^87^Sr/^86^Sr between rib and femur is very small (up to 0.0005) while the maximum differences between the petrous parts and the ribs and femora are 0.0032 and 0.0029 respectively (Fig 5). A similar observation can be done for [Sr] with a maximum difference of 34 ppm between ribs and femora and differences between petrous parts and ribs and femora of up to 99 and 65 ppm respectively (Fig 5). An additional observation lies in the fact that all petrous parts except that from individual 8 (who originates from Argentina) have a higher strontium isotope ratio than their respective femora and ribs (Fig 5). Furthermore, except in the case of Individual 10, when the difference in ^87^Sr/^86^Sr between rib and femur is > 0.0001 (Individuals 3, 7, 10, 13 and 14), [Sr] and ^87^Sr/^86^Sr of the femur falls between those of the respective petrous parts and ribs (Fig 6).

## Discussion

### Cremation conditions

The carbon isotope results confirm that the final carbon isotope composition of burned bone is heavily influenced by the fuel used (e.g., large amounts of wood in archaeological cremations compared to optimized amounts of gas (mostly natural gas [47]) in modern cremation retorts). Indeed, the carbon isotope ratios of the modern cremated human remains are similar to that of the modern cow tibia heated in a muffle furnace where no fuel was used rather than that of archaeological human remains for which large amounts of wood (or other fuel) was used during cremation and have very negative δ^13^C values (-31 to -23‰ [39,45]). The carbon isotope ratios of natural gas are also quite variable depending on its origin and composition and can be extremely variable with values going from -52 to -18‰ (e.g., [55]). This large variation could explain the larger range of values observed in the carbon isotope ratios of the bones burned in modern crematoria compared to archaeological bones (Fig 1). The shorter length (ca. 2h) and the closed settings of the modern cremation retort ([47]) compared to an open fire archaeological cremation that lasted between 4 and 8 hours suggest that, in the case of modern cremation, the organic carbon of the various tissues (i.e., skin, muscle, bone) represents an important pool of carbon that can exchange carbon with the carbon present in the carbonate fraction of bone apatite. That pool has lower carbon isotope ratios compared to bone apatite carbonates but still higher than in wood. In particular, in the US, high C4-diets are common, leading to even higher δ^13^C values in human tissues [56]. A study based on 197 18^th^–19^th^ century unburnt North American human remains showed a range of bone and dentine δ^13^C values ranging from -20.9 to -7.5‰ and an average offset in δ^13^C value of 5.5‰ between bone collagen and bone apatite carbonates [57]. The potential presence of clothing during cremation can also contribute some carbon but probably to a lesser extent as the clothes are likely to burn away well before the bones reach the temperature at which carbon exchanges can occur. Still, those clothes could slightly contribute to the final carbon isotope ratios of burned bone [45]. Clothes are often made of wool and cotton with isotope ratios between -27 and -24‰ [58,59]. The fact that most bone fragments exhibit δ^13^C values above -23‰ (80%) suggests that the clothing (and any potential coffin) had limited impact on the final carbon isotope ratios of burned bone. Instead the organic carbon pool seems to be the dominant input of carbon, with an influence of the natural gas used leading to a larger range of δ^13^C values but with overall higher values than in ancient cremations carried out on an open pyre. Bone density could also impact the carbon exchanges that occur during burning with the petrous part being denser and exchanging less, while ribs, being much more porous, seem to exchange more.

The expectation is that the petrous part of the temporal bone would exchange the least with the organic carbon of the body due to its location and high density. Furthermore, the head is unlikely to be covered by clothes. The ribs, on the other hand, are expected to exchange the most with the organic carbon and clothes. The ribs are much more porous and the rib cage is a large container of organic carbon with muscles, organs, etc. and is expected to be covered by one or even more layers of clothing. The femur, surrounded by muscles and a single layer of clothing, is expected to show intermediate exchanges of carbon. The carbon isotope results confirm this pattern (Fig 1). The differences in the carbon isotope ratios in the individuals burned in *Crematoria A* and *B* are more difficult to explain. During the period of cremation, protocols between facilities are relatively standardized in the US funerary industry. The difference could be linked to variation in cardboard coffins or lack of a cardboard coffin used at *Crematorium A* and *Crematorium B*. This information is unfortunately unavailable but clearly shows that the way in which a body is cremated has a significant impact on carbon isotope composition of burned bone.

The oxygen isotope ratios are much more homogenous between the different bone groups than carbon isotope ratios. They are also closer to the δ^18^O values of the archaeological cremated human remains than the experimentally burned cow tibia (Fig 2). In the laboratory experiment, the cow tibia fragments were defleshed and heated in a small muffle furnace with no addition of oxygen [40,44] while in archaeological contexts, cremations are likely to have taken place outside (i.e., much more oxygen available) and with fully fleshed bodies. In modern crematoriums, full bodies are cremated with gas (which contained little amounts of oxygen) and, to avoid the production of smoke during modern cremations, the amount of air (i.e., oxygen) in the furnace is adjusted to ensure complete oxidation of the organic carbon of the body [60]. As such the amount of oxygen is likely to be more similar to an outdoor cremation than a closed muffle furnace. This shows that the oxygen isotope ratios of burned bone are heavily influenced by the amount of oxygen available during cremation and/or the presence of flesh. The low amounts of cyanamide detected (only three femur samples with CN/P > 0.10) also suggest that the bones were burned with sufficient amounts of oxygen to avoid reducing conditions that have been suggested to be the cause of the incorporation of cyanamide in burned bone. Ribs are very porous and thin which could explain the lack of cyanamide in them after burning but the fact that significant amounts of cyanamide were only found in femora and not in petrous parts that are both particularly dense warrants further investigation. In forensic contexts, δ^18^O values might help understand the circumstances of fire-affected bone as oxygen availability is different for an open compared to closed environment.

### Mobility and life-reconstruction

A recent study of modern humans (based on tooth enamel) from the Netherlands showed that 98% of the studied individuals had a ^87^Sr/^86^Sr between 0.7088 and 0.7099 [61]. A similar study in Columbia showed a slightly larger range of values with a mean ^87^Sr/^86^Sr of 0.7081 ± 0.0035 (2SD; n = 156 [62]). The results obtained here show that all but four bone fragments (9.5% of the samples) fall between 0.7088 and 0.7102 which is a very similar range to that observed in the Netherlands. The single fragment with a value below 0.7086 is the petrous part of the individual born in Argentina. This is in agreement with low ^87^Sr/^86^Sr (as low as 0.7039) observed in small animals and plants in Argentina [63,64]. The three fragments with values higher than 0.7102 are also petrous parts with ^87^Sr/^86^Sr values of 0.7107, 0.7012 and 0.7130. The further observation that all petrous parts but one have higher ^87^Sr/^86^Sr compared to their respective ribs and femora was unexpected and warrants further investigations. A similar observation was done at the Early Medieval cremation cemetery of Echt in the Netherlands, where 8 out of 10 petrous parts have values higher than diaphysis [16], but no such trend was detected at the Late Bronze Age site of Inzersdorfs, Austria, where only 7 out of 15 petrous parts have higher values than the respective diaphysis [65].

**Fig 7:**
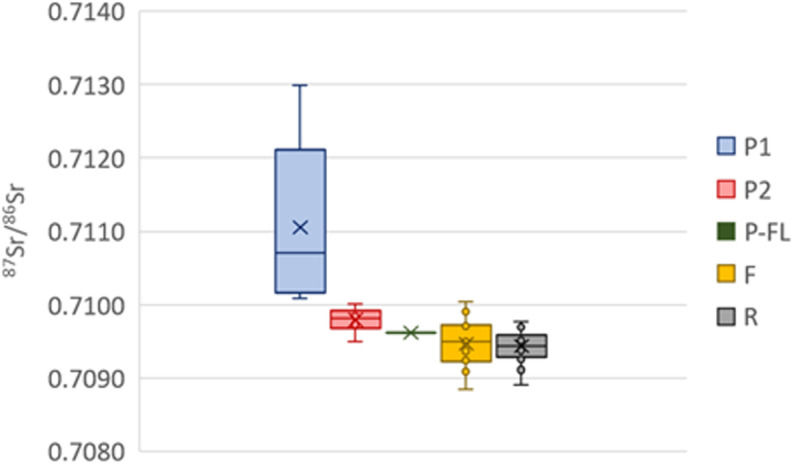
Strontium isotope ratios of the petrous parts (P1, P2, P-FL), femora (F) and ribs (R).

Comparing the values measured on the petrous parts with the strontium isoscape of the US based on river waters and averaged within watersheds [66], a clear pattern can be observed. The five individuals with values above 0.7100 belong to individuals born in areas with higher strontium isotope ratios (Group P1 – Fig 7) compared to the six individuals with values below or equal to 0.7100 that were all born in Illinois or Michigan (Group P2 – Fig 7). The individual with the highest value (0.7130) was born very close to an area with very high strontium isotope ratios. Such trends are not observed for the femora and ribs that all have values between 0.7088 and 0.7100. This range is observed in many parts of the world in most carbonate and some recent sedimentary rocks. Still, that all bone fragments reflecting dietary intake during adulthood fall within a similar range and that the differences between rib and femur are lower than 0.0005 for every single individual shows that the strontium isotope range in the foods consumed by those living in the US (or at least in the East of the US) is quite homogenous but that it might not have been the case 50 to 80 years ago. The fact that this range (0.7088–0.7100) is almost identical to that observed in modern humans from the Netherlands (0.7088–0.7099 [61]) further supports the hypothesis of a “global” signal not only in the eastern US but also across different countries and even continents. Unfortunately, this would limit the potential of using ^87^Sr/^86^Sr to identify the origin of individuals born recently in the US and only the petrous parts of older individuals would provide useful results.

If it can indeed be assumed that food became much more homogenous across the US in the last fifty years, we can argue that ^87^Sr/^86^Sr of the individuals studied here changed during their lifetime from a “local/childhood” signal to a “global” signal regardless of where they moved to. This will, however, be hard to observe in individuals from group P2 as their “local/childhood” signal (0.7095–0.7100) overlaps with the “global” one (0.7088–0.7100). The same can be said for the individual born in the UK whose petrous part has a strontium isotope ratio of 0.7097. The individual born in Argentina, however, shows a shift from a low value of 0.7086 to the “global” one. The strontium concentrations vary between 55 and 173 ppm suggesting a diet mostly based on terrestrial resources. Still, they seem to be more variable than the ^87^Sr/^86^Sr with differences of up to 34 ppm between rib and femur. A change in diet (e.g., becoming vegetarian or vegan) might have a significant impact on [Sr] and could explain the differences observed in [Sr] between ribs and femurs even when ^87^Sr/^86^Sr remains unchanged (e.g., individuals 8 and 11 – Fig 6).

These results also highlight an important point that requires further investigation: the strontium turnover rates of different skeletal elements. The large differences in ^87^Sr/^86^Sr observed between different pieces of the temporal bone (up to 0.0015) confirms that only the petrous part of the temporal bone does not remodel and retains a strontium isotope ratio relating to childhood. Therefore, extreme care is needed when sampling. In this work, while the sampling was done with care, it may not have been optimal and some bone that did remodel might have been sampled. Since this research was carried out, Veselka et al. (2021b) developed an optimal sampling method. Furthermore, while it is clear that the non-remodeling inner cortex of petrous part of the temporal bone reflects a signal related to early childhood while the strontium isotope ratios of ribs and femur represents an average of several years or decades of strontium intake prior to death, the extent to which ribs and femur represent different periods of one’s life remains vague and most likely depend on the age of the individuals (here most individuals were old, between 47 and 85 years). Our results seem, nevertheless, to confirm that ribs represent fewer years than femora as [Sr] and ^87^Sr/^86^Sr of four out of five femora with Δ^87^Sr/^86^Sr_(rib-femur)_ > 0.0001 fall between those of the ribs and the petrous parts (Fig 6). However, the sample size is very small and in the nine other cases, the difference between ribs and femora is negligible (< 0.0001).

## Conclusion

The multi-isotopic study of different skeletal elements of modern cremated humans who died in the US between 2010 and 2016 allowed us to improve our understanding of cremation practices and assess the limitations of strontium isotope analyses in forensic contexts. It becomes clear from these results that the way in which the cremation is performed (outdoor pyre vs. cremation retort) is the dominant factor impacting the carbon isotope ratios of calcined bone apatite carbonates while oxygen availability during burning seems to significantly impact the oxygen isotope ratios. The differences observed in the infrared and carbon and oxygen isotope results between different skeletal elements clearly shows how variable a cremation is and that multiple skeletal elements should be analyzed when reconstructing funerary practices. These results offer a chance to better understand ancient cremation parameters such as position of the body on the pyre, size of the pyre, oxygen availability, etc.

The strontium concentrations and isotope ratios measured in the fourteen individuals highlight the potentials and the limitations of such studies on modern individuals. Petrous parts of the temporal bone clearly reflect a signal of childhood diet and could be linked to the geographical place of birth, whereas ribs and femora reflect the later years/decades of an individual’s life. It seems, however, that globalization over the last 50 years limits the use of strontium isotope ratios for provenance studies of modern individuals based on bones other than petrous parts. Indeed, regardless of their place of death (and residence beforehand), all ribs and femora have values between 0.7088 and 0.7100, which does not at all represent the variations in biologically available strontium isotope ratios in the US. This probably explains the small differences observed between rib and femur and more research should be carried out on individuals that died more than 50 years ago to gather more information about the differences in strontium turnover rates between these and other skeletal elements. Still, the results on the petrous parts are very exciting as they show that, for historical and archaeological periods in the US, the use of strontium isotope ratios can be particularly powerful predictors of geographical origin.

## References

[1] Lanting JN, Brindley AL. Dating cremated bone: the dawn of a new era. The Journal of Irish Archaeology. 1998;1(1):1–7.

[2] Harvig L, Frei KM, Price TD, Lynnerup N. Strontium isotope signals in cremated petrous portions as indicator for childhood origin. PLoS One. 2014;9(7):e101603. doi: 10.1371/journal.pone.0101603 25010496 PMC4091946

[3] Snoeck C, Lee-Thorp J, Schulting R, de Jong J, Debouge W, Mattielli N. Calcined bone provides a reliable substrate for strontium isotope ratios as shown by an enrichment experiment. Rapid Commun Mass Spectrom. 2015;29(1):107–14. doi: 10.1002/rcm.7078 25462370

[4] Dalle S, Snoeck C, Sengeløv A, Salesse K, Hlad M, Annaert R, et al. Strontium isotopes and concentrations in cremated bones suggest an increased salt consumption in Gallo-Roman diet. Sci Rep. 2022;12(1):9280. doi: 10.1038/s41598-022-12880-4 35660749 PMC9166795

[5] Snoeck C, Pouncett J, Ramsey G, Meighan IG, Mattielli N, Goderis S, et al. Mobility during the neolithic and bronze age in northern ireland explored using strontium isotope analysis of cremated human bone. Am J Phys Anthropol. 2016;160(3):397–413. doi: 10.1002/ajpa.22977 27061584

[6] Snoeck C, Pouncett J, Claeys P, Goderis S, Mattielli N, Parker Pearson M, et al. Strontium isotope analysis on cremated human remains from Stonehenge support links with west Wales. Sci Rep. 2018;8(1):10790. doi: 10.1038/s41598-018-28969-8 30072719 PMC6072783

[7] Snoeck C, Jones C, Pouncett J, Goderis S, Claeys P, Mattielli N, et al. Isotopic evidence for changing mobility and landscape use patterns between the Neolithic and Early Bronze Age in western Ireland. Journal of Archaeological Science: Reports. 2020;30:102214. doi: 10.1016/j.jasrep.2020.102214

[8] Snoeck C, Capuzzo G, Veselka B, Salesse K, Kontopoulos I, Annaert R, et al. Unravelling the mysteries hidden within the cremated human remains from Belgium - The interdisciplinary CRUMBEL project. InBeyond urnfields New perspectives on Late Bronze Age - Early Iron Age funerary practices in northwest Europe. 2023;16:271–87. Verlag ludwig.

[9] Cavazzuti C, Bresadola B, d’Innocenzo C, Interlando S, Sperduti A. Towards a new osteometric method for sexing ancient cremated human remains. Analysis of Late Bronze Age and Iron Age samples from Italy with gendered grave goods. PLoS One. 2019;14(1):e0209423. doi: 10.1371/journal.pone.0209423 30699127 PMC6353077

[10] Cavazzuti C, Cardarelli A, Quondam F, Salzani L, Ferrante M, Nisi S, et al. Mobile elites at Frattesina: flows of people in a Late Bronze Age ‘port of trade’ in northern Italy. Antiquity. 2019;93(369):624–44. doi: 10.15184/aqy.2019.59

[11] Grupe G, Klaut D, Mauder M, Kröger P, Lang A, Mayr C, et al. Multi‐isotope provenancing of archaeological skeletons including cremations in a reference area of the European Alps. Rapid Communications in Mass Spectrometry. 2018;32(19):1711–27. 29949218 10.1002/rcm.8218

[12] Sebald S, Zeiler M, Grupe G. Provenance analysis of human cremations by 87Sr/86Sr isotopic ratios: migration into an Iron Age mining region in North-Rhine Westphalia. Open Journal of Archaeometry. 2018;4(1).

[13] Price T, Arcini C, Gustin I, Drenzel L, Kalmring S. Isotopes and human burials at Viking Age Birka and the Mälaren region, east central Sweden. Journal of Anthropological Archaeology. 2018;49(1):19–38.

[14] Graham D, Bethard J. Reconstructing the origins of the Perrins Ledge cremains using strontium isotope analysis. Journal of Archaeological Science: Reports. 2019;24(1):350–62.

[15] Taylor N, Frei K, Frei R. A strontium isotope pilot study using cremated teeth from the Vollmarshausen cemetery, Hesse, Germany. Journal of Archaeological Science: Reports. 2020;31:102356.

[16] Veselka B, Capuzzo G, Annaert R, Mattielli N, Boudin M, Dalle S, et al. Divergence, diet, and disease: the identification of group identity, landscape use, health, and mobility in the fifth- to sixth-century AD burial community of Echt, the Netherlands. Archaeol Anthropol Sci. 2021;13(6). doi: 10.1007/s12520-021-01348-7

[17] Sabaux C, Veselka B, Capuzzo G, Snoeck C, Sengeløv A, Hlad M, et al. Multi-proxy analyses reveal regional cremation practices and social status at the Late Bronze Age site of Herstal, Belgium. Journal of Archaeological Science. 2021;132:105437. doi: 10.1016/j.jas.2021.105437

[18] Kalenderian V, Snoeck C, Palstra S, Nowell G, Seif A. Migration and mobility in Roman Beirut: The isotopic evidence. Journal of Archaeological Science: Reports. 2023;49:104044.

[19] Löffelmann T, Snoeck C, Richards JD, Johnson LJ, Claeys P, Montgomery J. Sr analyses from only known Scandinavian cremation cemetery in Britain illuminate early Viking journey with horse and dog across the North Sea. PLoS One. 2023;18(2):e0280589. doi: 10.1371/journal.pone.0280589 36724154 PMC9891522

[20] Dalle S, Capuzzo G, Hlad M, Veselka B, Annaert R, Boudin M, et al. Hidden transitions. New insights into changing social dynamics between the Bronze and Iron Age in the cemetery of Destelbergen (Belgium). Journal of Archaeological Science: Reports. 2023;49:103979.

[21] Harbeck M, Schleuder R, Schneider J, Wiechmann I, Schmahl WW, Grupe G. Research potential and limitations of trace analyses of cremated remains. Forensic Sci Int. 2011;204(1–3):191–200. doi: 10.1016/j.forsciint.2010.06.004 20609539

[22] Sarancha JJ, Eerkens JW, Hopkins CJ, Gonçalves D, Cunha E, Oliveira-Santos I, Vassalo A, Gordon GW. The effects of burning on isotope ratio values in modern bone: Importance of experimental design for forensic applications. Forensic Science International. 2022;337:111370. 35816894 10.1016/j.forsciint.2022.111370

[23] Tuross N, Behrensmeyer AK, Eanes ED. Strontium increases and crystallinity changes in taphonomic and archaeological bone. Journal of Archaeological Science. 1989;16(6):661–72. doi: 10.1016/0305-4403(89)90030-7

[24] Budd P, Montgomery J, Barreiro B, Thomas RG. Differential diagenesis of strontium in archaeological human dental tissues. Applied Geochemistry. 2000;15(5):687–94. doi: 10.1016/s0883-2927(99)00069-4

[25] Kubat J, Nava A, Bondioli L, Dean MC, Zanolli C, Bourgon N, et al. Dietary strategies of Pleistocene Pongo sp. and Homo erectus on Java (Indonesia). Nat Ecol Evol. 2023;7(2):279–89. doi: 10.1038/s41559-022-01947-0 36646949

[26] Nava A, Lugli F, Romandini M, Badino F, Evans D, Helbling AH, et al. Early life of Neanderthals. Proc Natl Acad Sci U S A. 2020;117(46):28719–26. doi: 10.1073/pnas.2011765117 33139541 PMC7682388

[27] Lugli F, Nava A, Sorrentino R, Vazzana A, Bortolini E, Oxilia G, Silvestrini S, Nannini N, Bondioli L, Fewlass H, Talamo S. Tracing the mobility of a Late Epigravettian (~ 13 ka) male infant from Grotte di Pradis (Northeastern Italian Prealps) at high-temporal resolution. Scientific reports. 2022 May 16;12(1):8104. 35577834 10.1038/s41598-022-12193-6 PMC9110381

[28] Pearson MP, Chamberlain A, Jay M, Marshall P, Pollard J, Richards C, et al. Who was buried at Stonehenge?. Antiquity. 2009;83(319):23–39. doi: 10.1017/s0003598x00098069

[29] Montgomery J. Passports from the past: Investigating human dispersals using strontium isotope analysis of tooth enamel. Ann Hum Biol. 2010;37(3):325–46. doi: 10.3109/03014461003649297 20367186

[30] Burton JH, Price TD, Middleton WD. Correlation of Bone Ba/Ca and Sr/Ca due to Biological Purification of Calcium. Journal of Archaeological Science. 1999;26(6):609–16. doi: 10.1006/jasc.1998.0378

[31] Sillen A, Kavanagh M. Strontium and paleodietary research: A review. Am J Phys Anthropol. 1982;25(S3):67–90. doi: 10.1002/ajpa.1330250505

[32] Hess J, Bender ML, Schilling JG. Evolution of the ratio of strontium-87 to strontium-86 in seawater from cretaceous to present. Science. 1986;231(4741):979–84. doi: 10.1126/science.231.4741.979 17740296

[33] Schulting R, Snoeck C, Loe L, Gilmour N. Strontium isotope analysis of the Mesolithic cremation from Langford, Essex, England. Mesolithic Miscellany. 2016;24(1).

[34] Hedges REM, Clement JG, Thomas CDL, O’connell TC. Collagen turnover in the adult femoral mid-shaft: modeled from anthropogenic radiocarbon tracer measurements. Am J Phys Anthropol. 2007;133(2):808–16. doi: 10.1002/ajpa.20598 17405135

[35] Robins SP, New SA. Markers of bone turnover in relation to bone health. Proc Nutr Soc. 1997;56(3):903–14. doi: 10.1079/pns19970098 9483659

[36] Veselka B, Locher H, de Groot JCMJ, Davies GR, Snoeck C, Kootker LM. Strontium isotope ratios related to childhood mobility: Revisiting sampling strategies of the calcined human pars petrosa ossis temporalis. Rapid Commun Mass Spectrom. 2021;35(7):e9038. doi: 10.1002/rcm.9038 33370492

[37] Zazzo A, Saliège J-F, Lebon M, Lepetz S, Moreau C. Radiocarbon Dating of Calcined Bones: Insights from Combustion Experiments Under Natural Conditions. Radiocarbon. 2012;54(3–4):855–66. doi: 10.1017/s0033822200047500

[38] Zazzo A, Lebon M, Chiotti L, Comby C, Delqué-Količ E, Nespoulet R, et al. Can we Use Calcined Bones for 14C Dating the Paleolithic?. Radiocarbon. 2013;55(3):1409–21. doi: 10.1017/s0033822200048347

[39] Snoeck C, Brock F, Schulting RJ. Carbon exchanges between bone apatite and fuels during cremation: impact on radiocarbon dates. Radiocarbon. 2014;56(2):591–602.

[40] Snoeck C, Schulting R, Lee-Thorp J, Lebon M, Zazzo A. Impact of heating conditions on the carbon and oxygen isotope composition of calcined bone. Journal of Archaeological Science. 2016;65(1):32–43.

[41] Thompson T, Gauthier M, Islam M. The application of a new method of Fourier Transform Infrared Spectroscopy to the analysis of burned bone. Journal of Archaeological Science. 2009;36(3):910–4.

[42] Thompson TJU, Islam M, Bonniere M. A new statistical approach for determining the crystallinity of heat-altered bone mineral from FTIR spectra. Journal of Archaeological Science. 2013;40(1):416–22. doi: 10.1016/j.jas.2012.07.008

[43] Lebon M, Reiche I, Bahain J-J, Chadefaux C, Moigne A-M, Fröhlich F, et al. New parameters for the characterization of diagenetic alterations and heat-induced changes of fossil bone mineral using Fourier transform infrared spectrometry. Journal of Archaeological Science. 2010;37(9):2265–76. doi: 10.1016/j.jas.2010.03.024

[44] Snoeck C, Lee-Thorp J, Schulting R. From bone to ash: Compositional and structural changes in burned modern and archaeological bone. Palaeogeography, palaeoclimatology, palaeoecology. 2014;416:55–68.

[45] Salesse K, Stamataki E, Kontopoulos I, Verly G, Annaert R, Boudin M, et al. These boots are made for burnin’: Inferring the position of the corpse and the presence of leather footwears during cremation through isotope (δ13C, δ18O) and infrared (FTIR) analyses of experimentally burnt skeletal remains. PLoS One. 2021;16(10):e0257199. doi: 10.1371/journal.pone.0257199 34644308 PMC8513878

[46] Snoeck C. Isotope analysis from cremated remains. Burnt human remains: Recovery, analysis, and interpretation. 2023:273–89.

[47] Schultz JJ, Warren MW, Krigbaum JS. Analysis of human cremains: Gross and chemical methods. The Analysis of Burned Human Remains. 2008:75–viii. doi: 10.1016/b978-012372510-3.50006-x

[48] McMillan R, Snoeck C, de Winter NJ, Claeys P, Weis D. Evaluating the impact of acetic acid chemical pre-treatment on ‘old’ and cremated bone with the ‘Perio-spot’ technique and ‘Perios-endos’ profiles. Palaeogeography, Palaeoclimatology, Palaeoecology. 2019;530:330–44.

[49] de Winter NJ, Snoeck C, Claeys P. Seasonal Cyclicity in Trace Elements and Stable Isotopes of Modern Horse Enamel. PLoS One. 2016;11(11):e0166678. doi: 10.1371/journal.pone.0166678 27875538 PMC5119779

[50] Weis D, Kieffer B, Maerschalk C, Barling J, De Jong J, Williams G, et al. High‐precision isotopic characterization of USGS reference materials by TIMS and MC‐ICP‐MS. Geochemistry, Geophysics, Geosystems. 2006;7(8).

[51] Royston P. Remark AS R94: A remark on algorithm AS 181: The W-test for normality. Journal of the Royal Statistical Society Series C (Applied Statistics). 1995;44(4):547–51.

[52] Myles H, Wolfe DA, Chicken E. Nonparametric statistical methods. Ed. John Wiley and Sons. New York, NY. 1973;503.

[53] Snoeck C, Beasley M, Steadman D. Infrared and isotope data from cremated human remains. 2024. doi: 10.5281/zenodo.11516688

[54] Stamataki E, Kontopoulos I, Salesse K, McMillan R, Veselka B, Sabaux C, et al. Is it hot enough? A multi-proxy approach shows variations in cremation conditions during the Metal Ages in Belgium. Journal of Archaeological Science. 2021;136:105509.

[55] Zou Y-R, Cai Y, Zhang C, Zhang X, Peng P. Variations of natural gas carbon isotope-type curves and their interpretation – A case study. Organic Geochemistry. 2007;38(8):1398–415. doi: 10.1016/j.orggeochem.2007.03.002

[56] Jahren AH, Kraft RA. Carbon and nitrogen stable isotopes in fast food: signatures of corn and confinement. Proc Natl Acad Sci U S A. 2008;105(46):17855–60. doi: 10.1073/pnas.0809870105 19001276 PMC2582047

[57] France CAM, Owsley DW. Stable Carbon and Oxygen Isotope Spacing Between Bone and Tooth Collagen and Hydroxyapatite in Human Archaeological Remains. Int J Osteoarchaeol. 2013;25(3):299–312. doi: 10.1002/oa.2300

[58] von Holstein ICC, Makarewicz CA. Geographical variability in northern European sheep wool isotopic composition (δ(13) C, δ(15) N, δ(2) H values). Rapid Commun Mass Spectrom. 2016;30(12):1423–34. doi: 10.1002/rcm.7578 27197035

[59] Saranga Y, Flash I, Paterson AH, Yakir D. Carbon isotope ratio in cotton varies with growth stage and plant organ. Plant Science. 1999;142(1):47–56. doi: 10.1016/s0168-9452(99)00004-7

[60] McKinley JI. Spong Hill Part VIII: The Cremations. East Anglian Archaeology 69, East Dereham, Norfolk. 1994;69.

[61] Kootker LM, Plomp E, Ammer ST, Hoogland V, Davies GR. Spatial patterns in 87Sr/86Sr ratios in modern human dental enamel and tap water from the Netherlands: implications for forensic provenancing. Science of the Total Environment. 2020;729:138992. 32361454 10.1016/j.scitotenv.2020.138992

[62] Castellanos D, DiGangi EA, Bethard JD, Kamenov G, González-Colmenares G, Sanabria-Medina C. Assessment of carbon, oxygen, strontium, and lead isotopic variation in modern Colombian teeth: An application to human identification. J Forensic Sci. 2023;68(6):1856–74. doi: 10.1111/1556-4029.15372 37646362

[63] Barberena R, Tessone A, Cagnoni M, Gasco A, Durán V, Winocur D, et al. Bioavailable strontium in the Southern Andes (Argentina and Chile): A tool for tracking human and animal movement. Environmental Archaeology. 2021;26(3):323–35.

[64] Serna A, Prates L, Mange E, Salazar-García DC, Bataille CP. Implications for paleomobility studies of the effects of quaternary volcanism on bioavailable strontium: A test case in North Patagonia (Argentina). Journal of Archaeological Science. 2020;121:105198. doi: 10.1016/j.jas.2020.105198

[65] Fritzl M, Waltenberger L, James HF, Snoeck C, Rebay-Salisbury K. Over the river and into the hills: locals and non-locals at Inzersdorf, a late Bronze Age cemetery in the Traisen Valley (Austria). Archaeol Anthropol Sci. 2024;16(9). doi: 10.1007/s12520-024-02054-w

[66] Bataille CP, Bowen GJ. Mapping 87Sr/86Sr variations in bedrock and water for large scale provenance studies. Chemical Geology. 2012;304:39–52.

